# Review: Impact of Helminth Infection on Antimycobacterial Immunity—A Focus on the Macrophage

**DOI:** 10.3389/fimmu.2017.01864

**Published:** 2017-12-22

**Authors:** Roland Lang, Judith Schick

**Affiliations:** ^1^Institute of Clinical Microbiology, Immunology and Hygiene, Universitätsklinikum Erlangen, Friedrich-Alexander Universität Erlangen-Nürnberg, Erlangen, Germany

**Keywords:** helminth infection, tuberculosis, vaccination, type 2 immune response, macrophage, IL-4, IL-10

## Abstract

Successful immune control of *Mycobacterium tuberculosis* (MTB) requires robust CD4^+^ T cell responses, with IFNγs as the key cytokine promoting killing of intracellular mycobacteria by macrophages. By contrast, helminth infections typically direct the immune system toward a type 2 response, characterized by high levels of the cytokines IL-4 and IL-10, which can antagonize IFNγ production and its biological effects. In many countries with high burden of tuberculosis, helminth infections are endemic and have been associated with increased risk to develop tuberculosis or to inhibit vaccination-induced immunity. Mechanistically, regulation of the antimycobacterial immune response by helminths has been mostly been attributed to the T cell compartment. Here, we review the current status of the literature on the impact of helminths on vaccine-induced and natural immunity to MTB with a focus on the alterations enforced on the capacity of macrophages to function as sensors of mycobacteria and effector cells to control their replication.

## Immunological Requirements for Effective Control of *Mycobacterium tuberculosis* (MTB) Infection

### Innate Recognition of MTB

The innate immune system detects incoming mycobacteria during phagocytosis by alveolar macrophages in the lung. The hydrophobic mycobacterial cell wall contains a large number of lipids, glycolipids, and lipoglycans that act as pathogen-associated molecular patterns (PAMPs), which are recognized by several classes of pattern recognition receptors (PRRs) [for review, see Ref. (1)]. Due to the intracellular lifestyle of MTB, which persists and replicates in the phagosome, endosomal PRR have ample opportunity to interact with mycobacterial ligands released into this compartment, e.g., DNA and RNA. With increasing time spent in its host cell, mycobacterial products and even the bugs themselves can enter the cytosol (2), where yet other PRRs sense the presence of intruding microbes.

**Figure 1:**
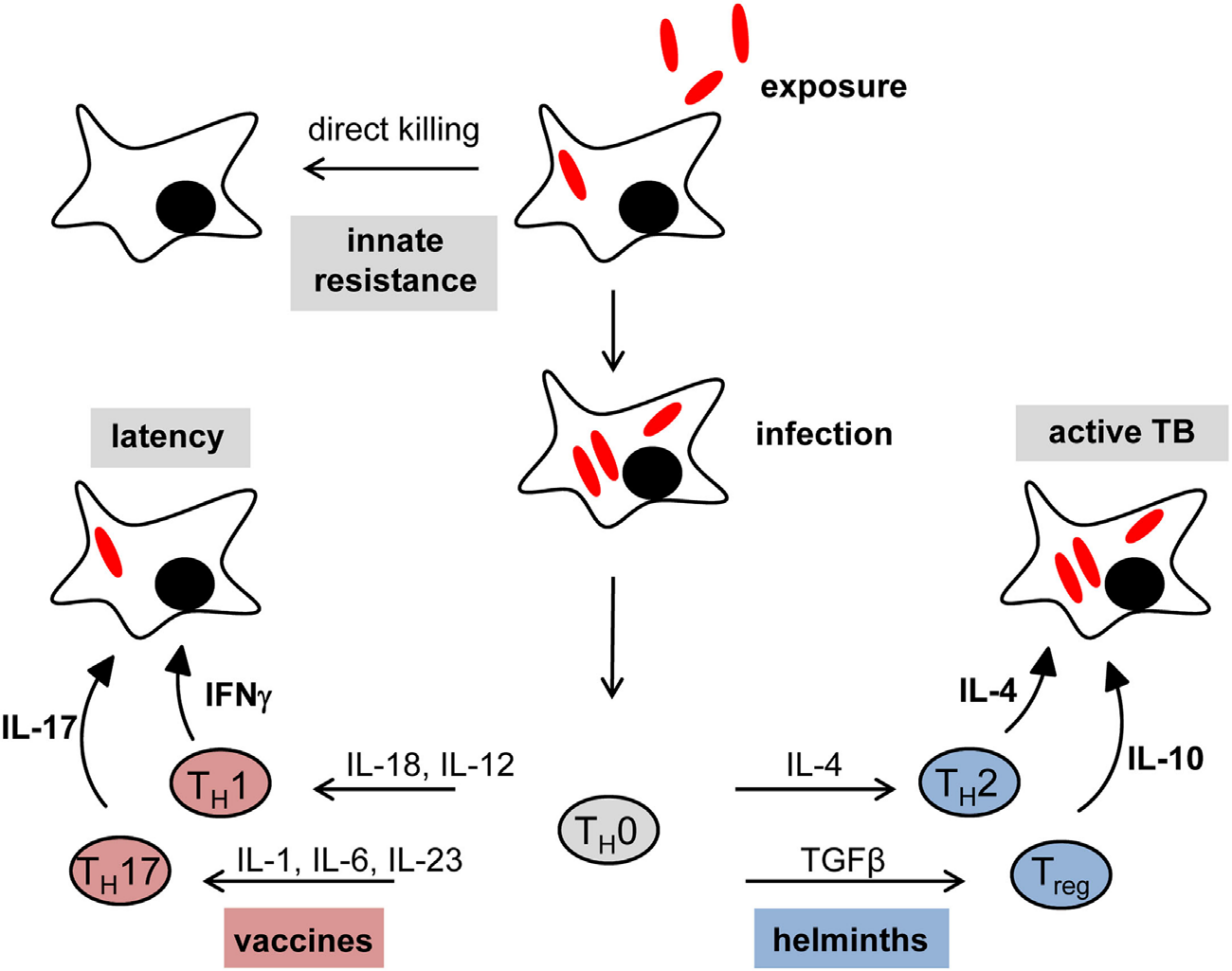
Immune checkpoints in tuberculosis: impact of vaccination and helminth infection. In many cases, the initial exposure to mycobacteria results in direct killing by alveolar macrophages without the need for an adaptive immune response (innate resistance). Failure of initial innate control mechanisms leads to primary infection. The set of chemokines and cytokines produced by innate immune cells are crucial for the shaping of an effective adaptive immune response. The Th1 key cytokine IFNγ is necessary to establish and maintain latent infection. Moreover, IL-17 was found to be important for vaccination-induced protection against tuberculosis. However, concomitant helminth infection shifts the immune system toward a T helper type 2 (Th2)/regulatory T cells (Treg) response rather than a protective Th1/Th17 immune status, which leads to a higher risk to develop active disease and interferes with successful vaccination responses.

This initial interaction between macrophages and MTB is crucial: if the macrophage is able to kill MTB at this stage, no infection occurs and there is no need to call in adaptive immunity (Figure 1 “innate resistance”). Based on studies on transmission of MTB to household contacts measuring tuberculin skin test or quantiferon responses, this may be the situation in more than 50% of all exposures (3–5). However, since it is difficult to determine the true exposure of household contacts of patients with open tuberculosis to infectious aerosol, the percentage of innate resistance to MTB in humans could also be considerably lower (6). On the other hand, the finding that tuberculin skin test negativity in humans is linked to a chromosomal region overlapping the TNF1 locus provides evidence for genetic control of innate resistance to MTB infection (7). Clearly, the factors determining the initial fate of mycobacteria after ingestion by alveolar macrophages are very incompletely understood, and may range from cytokines such as TNF to antimicrobial peptides, the autophagy machinery and control of phagosomal maturation (8). Since all these macrophage functional processes are under the influence of signaling emanating from PRR, it makes sense to assume that the recognition of MTB by different PRR contributes to the initial decision if ingested bacilli survive or are killed. If the mycobacteria manage to establish an intracellular niche in the macrophage, the nature of the innate response (mostly the composition of chemokines and cytokines secreted) depends on PRR pathways and determines the type of adaptive immunity and the swiftness of a protective response characterized by robust Th1 and Th17 T cells.

Toll-like receptors (TLR) have been most intensively studied for their role in the response to mycobacteria. TLR2 and TLR4 bind to mycobacterial cell wall components lipoarabinomannan (LAM) and phosphatidylinositol mannosides (PIM), and lipomannan, respectively (9–12). The 19-kDa lipopeptide of MTB is also a TLR2 ligand (12). The endosomal TLR7 and TLR8 (the later only in humans, but not in mice) sense single-stranded RNA (13), while CpG-rich DNA was initially purified as the immunostimulatory principle of Bacille Calmette–Guerin (BCG) treatment and later explained by activation of TLR9 (14). Independent of their localization on the cell surface or in the phagosome, TLR2, TLR7/8, and TLR9 require the adapter protein Myd88 to activate gene expression. Myd88-dependent signaling is essential for host defense against experimental MTB infection in mice; however, as even the triple knockout of TLR2, TLR4, and TLR9 in mice does not increase mycobacterial load (15), the phenotype of Myd88^−/−^ mice is likely due to a lack of IL-1 receptor signaling rather than TLR activation (16–18).

More recently, several C-type lectins receptor (CLR) have been identified as receptors for PAMPs present in the mycobacterial cell wall [reviewed in Ref. (19)]. Dectin-1 is triggered by an unknown mycobacterial ligand and induces IL-12 production from DC (20) and collaborates with TLR2 in induction of cytokine gene expression (21). The genes for the CLR Mincle (Clec4e), Mcl (Clec4d), and Dectin-2 (Clec4n) are located in close vicinity in the genome and are referred to as the Dectin-2 family (22). Mincle is the receptor for the so-called mycobacterial cord factor trehalose-6,6′-dimycolate (TDM), the most abundant glycolipid in the cell wall of MTB (23, 24). Mincle can form dimers with the related CLR Mcl (25), and both receptors mutually enhance their cell surface protein levels (26). Dectin-2 binds mannose-rich fungal ligands (27, 28), but also the TLR2 ligand mannose-capped LAM (29). Most recently, DCAR (Clec4b1), another FcRγ-coupled CLR, was revealed to bind to PIM of the cell wall, to induce MCP-1 expression by macrophages, and to induce Th1 responses (30). All of these CLR signal *via* the Syk-Card9-Bcl10-Malt1 pathway to activate NFκB and upregulate expression of multiple chemokines, cytokines and inflammatory mediators causing inflammation and directing developing adaptive immune responses (31, 32). Knockout mice for Card9 are highly susceptible to challenge with MTB, with increased bacterial burden, uncontrolled granulocytic inflammation, and early death (33). The phenotype of mice deficient in individual CLR in MTB infection is much more moderate, as demonstrated for Mincle (34–36), Mcl (37), or shows no difference as in the case of Dectin-1 (38). Thus, similar to the case of TLR, there appears to be considerable redundancy of individual CLR for recognition of mycobacteria, but the combined ablation of the CLR response in Card9-deficient mice is detrimental and suggests an important function of this class of receptors in antimycobacterial defense.

In addition to TLR and CLR localized to the plasma membrane or the phagosome, NOD2 is a cytosolic sensor of mycobacterial muramyl dipeptide, which induces autophagy and activates NFκB-dependent gene expression (39). The ESX-1 secretion system of MTB generates phagosomal perforation and leakage of mycobacterial DNA and RNA. Binding of cytosolic DNA by the sensor cGAS leads to production of cyclic dinucleotides binding and activating STING, which triggers IFNβ expression in an IRF3-dependent manner (40, 41). The activation of the AIM2-NLRP3 inflammasome by cytosolic DNA is an additional pathway triggered by the intracellular pathogen MTB, and causes production of IL-1β (42, 43).

### IFNγ As Key Cytokine—Necessary but Not Sufficient

The single most important immunological molecule in defense against mycobacterial infection is the cytokine IFNγ (Figure 1 “latency”). Produced mostly by Th1 CD4^+^ T cells but also CD8^+^ T cells, NK cells and NKT cells, it acts on many different cell types by activating the Jak2-Stat1 signaling pathway that drives the expression of antimicrobial genes such as Nos2 (producing NO), IFNγ-induced GTPase-binding proteins of the 65 and 47 kDa families, bactericidal peptides (e.g., cathelicidin), and many more (44). The inability to upregulate IFNγ expression (e.g., deficiency in the cytokines IL-12 or IL-18, or their receptors), produce it, or respond to it (e.g., deficiency in the IFNγ receptor chains, Jak2 or Stat1), leads to high susceptibility to experimental mycobacterial infection in mouse models and in humans (45) (Figure 1 “active TB”).

While IFNγ clearly is necessary for a successful host response to infection, several studies have shown that it is not sufficient, as increased levels of IFNγ were indicative of disease progression rather than protection in mice and humans (46, 47). Beyond a simple lack of correlation between IFNγ levels and protection in MTB infection, there is even evidence that IFNγ production has to be tightly controlled to prevent damage to the host, particularly the lung tissue, during infection (48).

### Vaccination Responses—What Should We Look For?

Despite the nearly 100 years of vaccination with the *Mycobacterium bovis* strain BCG it is still very incompletely understood which vaccine-induced immune responses can be used as biomarkers for protective immunity (49). This applies to IFNγ, which is essentially required for successful immunity, but it is becoming increasingly clear that there is no linear relationship between vaccine-induced IFNγ and immune protection (48). More recently, IL-17-producing Th17 cells have been described as key players in vaccine-induced protection (50). Interestingly, the recombinant BCG vaccine VPM02 (ΔureC; hly^+^), which is more effective than the parental BCG in protecting against pulmonary tuberculosis in animal models, induces a balanced Th1/Th17 response and caused earlier recruitment of CD4^+^ T cells into the lungs after challenge infection (51). In the last years, there has been an increasing appreciation of the important role played by the route of immunization: Aguilo et al. demonstrated that BCG delivered intranasally protects DBA/2 mice against pulmonary tuberculosis much better than after subcutaneous immunization (52). An alternative strategy to improved BCG or novel recombinant MTB live vaccines is the development of subunit vaccines using recombinant MTB proteins. To be effective in generating robust antigen-specific CD4^+^ T cell responses, these protein vaccines require a carrier (e.g., liposomes) and adjuvants that activate antigen-presenting cells of the innate immune system for provision of costimulation and cytokines directing the desired Th cell differentiation signals. Here, the TLR9 ligands CpG ODN and IC31 are potent inducers of Th1 cells by triggering IL-12 release from DC and macrophages, and have therefore been used experimentally and in clinical studies (53–55). In contrast, ligands for certain CLR are potent inducers of Th17 differentiation: the Dectin-1 ligand Curdlan, a β-glucan, and the Mincle ligand TDB, an analog of the mycobacterial cord factor TDM, trigger production of IL-6, IL-23, and IL-1 from DC and macrophages, thereby providing robust differentiation signals toward a Th17 bias (24, 56, 57). The TDB-containing liposomal adjuvant CAF01 has been used in several experimental infection models, induces long-lived protective CD4 memory T cells in mice, and also appears to be effective in humans (58–61).

## Impact of Helminth Infection on Immunity to Mycobacteria

### The Epidemiological Evidence

Globally, more than two billion people are infected with parasitic helminths. Intestinal nematodes (*Ascaris lumbricoides*, hookworms, *Trichuris trichiura, Strongyloides stercoralis*), filaria (*Wuchereria bancrofti, Onchocerca volvulus, Loa loa*), and trematodes (*Schistosoma mansoni*) are the most frequent and important human worm infections (62). Similar to tuberculosis, helminth infections are often chronic and not acutely life-threatening. Helminths have complex life cycles and various developmental stages, and elicit distinct host immune reactions. Despite significant differences between specific helminth infections, the immune response to helminths is generally characterized by a T helper type 2 (Th2) pattern with high levels of the cytokines IL-4, IL-5, IL-9, IL-10, and IL-13, as well as eosinophilia, goblet and mast cell hyperplasia, and IgE-biased antibody isotype switching (62). Thus, helminth infection and tuberculosis share the chronic nature, but represent two extremes of immunological bias and immune effector mechanisms. Since tuberculosis and helminth infections are coinciding in many parts of the world, most notably in Africa, South America, and Asia, the possibility that concomitant helminth infection affects antimycobacterial immunity has been investigated by epidemiological studies.

### Helminth Infection and Vaccination against Tuberculosis

The efficacy of the BCG vaccine shows large geographic variation, with reduced protection in African and Asian countries, where worm infections are more prevalent than in Europe and North America (63, 64). Deworming of helminth-infected vaccinees before administration of BCG led to increased IFNγ and IL-12 production, yet decreased TGFβ levels, suggesting that intestinal helminths impair the development of a Th1 response to BCG (65). In the mouse model, *S. mansoni* infection decreased protective efficacy of BCG against MTB challenge and the capacity to produce IFNγ by splenocytes (66). As BCG vaccination is usually done in neonates, effects of maternal helminth infection on vaccination responses in the offspring have been investigated in mice and humans. Maternal infection with helminths had a strong negative effect on induction of Th1 immunity in BCG-vaccinated offspring (67). In another study, hookworm-infected mothers had reduced IFNγ responses to MTB proteins, but their children showed rather increased IFNγ production (68). In addition, anthelmintic treatment during pregnancy did not alter the neonatal responses to vaccination including BCG (69). Thus, the literature is at present contradictory whether helminth coinfection is indeed a strong factor in the suboptimal response to BCG and whether anthelmintic treatment can overcome this hurdle.

### Impact of Helminth Infection on the Development and Course of Active Tuberculosis

Patients with active tuberculosis were found to be more often coinfected with helminths compared to controls (70, 71). In addition, coinfection with helminths was associated with more advanced disease in tuberculosis patients, coupled to reduced IFNγ but increased production of IL-10 (72). On the other hand, a recent study showed that asymptomatic helminth infection is indeed associated with type 2 immunity in tuberculosis patients, but led to a lower frequency of smear-positive sputum samples, i.e., reduced the risk to develop open cavitary disease (73). Reflecting this ambiguous state of the literature on the impact of helminth infection on the course of active tuberculosis, a treatment study with albendazole showed no change in the clinical course after 2 months of treatment (74).

## Mechanisms of Antimycobacterial Immune Regulation by Helminths and Helminth-Induced Type 2 Immunity

### Direct Regulation of Macrophage Function by Helminths

Macrophages encounter worm eggs and larval stages in tissues and therefore directly interact with helminths and their products. A number of studies have investigated how different helminth species and their products modulate macrophage activation state and responsiveness to other microbial stimuli. While these studies employed different helminths or helminth-derived products and a variety of readout parameters of macrophage activation, they in general described inhibitory or modulating effects on macrophages. ES-62, a secreted protein of the filarial nematode *Acanthocheilonema viteae* inhibits the production of IL-12, TNF, and IL-6 in response to LPS/IFNγ (75). Secreted filarial cystatins were found to be taken up by macrophages and to induce expression of IL-10, which was sensitive to inhibition of MAPK and was regulated by the phosphatase DUSP1 (76). Microfilariae of *Brugia malayi* induced a regulatory phenotype in human monocytes characterized by expression of IL-10 and PD-L1 (77). A *Fasciola hepatica* fatty acid binding protein exerted suppression of cytokine release and MAPK activation in response to the TLR4 ligand, and was found to bind to the co-receptor for LPS, CD14 (78). Similarly, the excretory/secretory products of the tapeworms *Hymenolepis diminuta* and *Spirometra erinaceieuropaei* inhibited the production of inflammatory cytokines such as TNF by macrophages (79, 80). Recently, Aira et al. observed that antigens prepared from *H. diminuta* and *Trichuris muris* inhibit phagolysosomal maturation and antigen presentation to CD4^+^ T cells in macrophages infected with MTB, with a direct negative effect on the control of mycobacterial survival in macrophages (81). It should be noted that inhibition of macrophage activation is not uniformly observed upon contact with helminths, e.g., antigens from *S. mansoni* showed the opposite effect, enhancing mycobacterial control and decreasing IL-10 production (81). While inhibiting production of TNF and IL-6 by TLR-triggered dendritic cells, schistosomal egg antigens (SEA) activated the Nlrp3 inflammasome and IL-1β production by activating the Dectin-2-FcRγ-Syk pathway (82).

### Helminth-Induced Th2/Regulatory T Cells (Treg) Bias of the T Cell Response

A reduced Th1 and Th17 immunity to mycobacterial antigens in patients with latent or active tuberculosis by coinfection with helminths was described in several studies (72, 83–85). By contrast, the frequency of Treg was consistently increased in coinfected patients (73, 86), together with the production of IL-10 (73), which also contributed to inhibition of Th1 differentiation (84). *In vitro* exposure of human PBMC to SEA increased the expression of IL-10 and IL-4 by CD4^+^ T cells, which in turn caused a block in phagolysosomal maturation in mycobacteria-infected macrophages (87). The Treg/Th2-biased immune deviation in coinfected patients was corrected by anthelmintic treatment, which restored Th1 cells and diminished Treg numbers (86), decreased the levels of IL-10 (74), or reduced Th2 but increased Th1/Th17 cytokines (88). These data indicate that the bias of antimycobacterial immunity toward Th2/Treg in helminth coinfection is reversible upon treatment.

### Type 2 Regulation of Myeloid Cells

#### Alternative Macrophage Activation by IL-4 Increases Bacterial Burden in MTB Challenge

**Figure 2:**
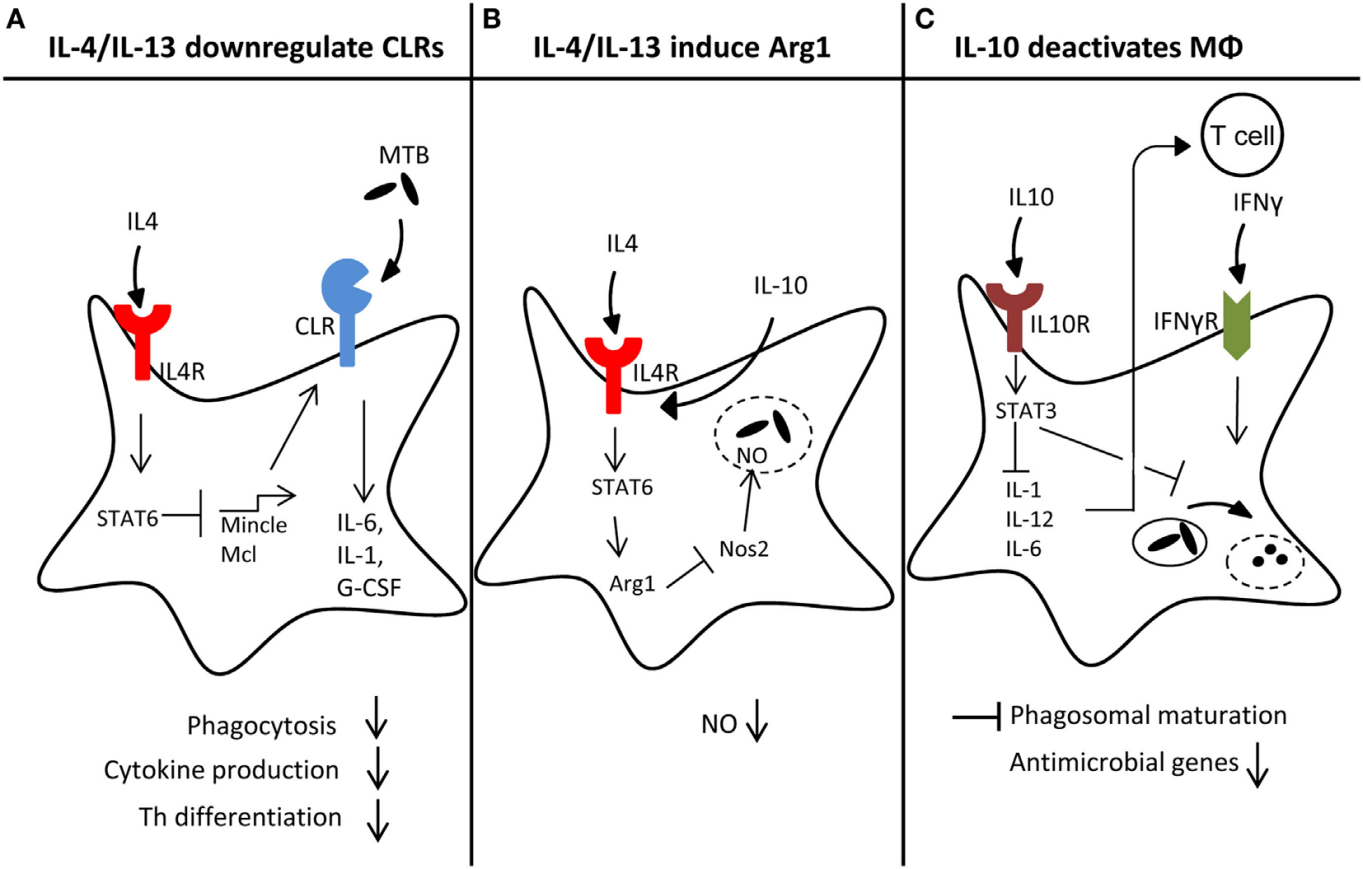
Macrophage reprogramming by helminth-induced type 2 responses. Helminth-induced type 2 responses can act on macrophages on different levels, thereby impeding antimicrobial responses toward *Mycobacterium tuberculosis* (MTB): **(A)** high levels of IL-4/IL-13 downregulate C-type lectin receptor (CLR) expression in a Stat6-dependent manner, which may lead to reduced amounts of pro-inflammatory cytokines like IL-6, IL-1, or G-CSF, impaired phagocytosis and Th1/Th17 differentiation. **(B)** Simultaneously, IL-4/IL-13 signaling through Stat6 strongly induces Arginase1 (Arg1), which counteracts Nos2 through substrate depletion, leading to reduced NO production and less effective killing of MTB inside the phagolysosome. **(C)** The anti-inflammatory cytokine IL-10, produced during helminth infection, can delay phagosomal maturation. Moreover, IL10R signaling through STAT3 inhibits IL-1, IL-12, or IL-6 expression, which in turn inhibits Th1 differentiation.

Experimental infections with filaria, schistosomes (89, 90), and the hookworm *Nippostrongylus brasiliensis* (91) caused a Th2-biased response to mycobacterial infection and, in part, higher bacterial burden. Potian et al. demonstrated that *Nippostrongylus* infection caused transiently increased mycobacterial burden through the activity of IL-4R-positive alternatively activated macrophages in the lung (91). While this study clearly demonstrated that the transfer of wild-type macrophages into IL-4R-deficient mice was sufficient to increase mycobacterial growth, the mechanistic basis has not been clarified in detail yet. One possible mechanism is upregulation of Arginase-1 expression, which is strongly induced by Stat6 signaling in response to IL-4/IL-13 signals in M2 macrophages and competes with iNOS for Arginine as a substrate (Figure 2B). Conditional Arginase-1 knockout mice lacking the enzyme in hematopoietic cells more efficiently clear MTB after pulmonary infection, correlating with enhanced NO production in the lung (92). However, in a coinfection model with MTB and *S. mansoni*, deletion of Arginase-1 did not alter the mycobacterial burden in coinfected mice, although it reversed lung pathology (93). Of note, infection with *N. brasiliensis* can also promote pulmonary Th1 cell responses and activation of alveolar macrophages, which was associated with enhanced control of early infection in a *M. bovis* BCG infection model (94). While these data appear to contradict those of Potian et al. (91), both studies agree in observing a reduced mycobacterial burden early in coinfection of *N. brasiliensis* and mycobacteria. Hence, timing of coinfection appears to be decisive for the impact of helminths on antimycobacterial immune responses.

#### Downregulation of TLR Expression in Helminth-Infected TB Patients

Babu et al. hypothesized that helminth infection may modulate immune responses by diminishing TLR expression and indeed identified reduced levels of TLR2 and TLR9 in PBMC of patients with filarial infection (95, 96). Cytokine responses to TLR2 and TLR9 ligands were decreased, suggesting that the impaired receptor expression was functionally relevant. Interestingly, anthelmintic treatment restored responsiveness to TLR ligands, indicating the reversibility of macrophage inhibition by filarial infection (95).

#### Downregulation of CLR Expression by IL-4 and IL-13

CLR are often expressed in cell type-specific and stimulus-dependent manners. Indeed, several CLR are used as markers for certain DC or macrophage cell types, e.g., Clec9a as marker for CD8^+^ DC. The cord factor receptor Mincle is strongly induced by stimulation of macrophages with the TLR ligands LPS or CpG but also by its ligand TDM itself (97, 98). Interestingly, mRNA expression of Mincle is high in monocytes and neutrophils in both mouse and human. However, during differentiation of human monocytes to DC *in vitro* in culture with GM-CSF plus IL-4, pronounced downregulation of Mincle mRNA was observed (99) and could be attributed to the action of IL-4 (100). This effect was also observed for the other Dectin-2 family CLR Mcl and Dectin-2, and was confirmed in mouse macrophages and DC at the mRNA and protein level by flow cytometry (100). By contrast, Dectin-1 expression was not affected negatively by IL-4. Both IL-4 and IL-13 impair expression of Mincle, and do so in a Stat6-dependent manner. Interestingly, the downregulation of Mincle and Mcl was associated with a reduced production of TNF and G-CSF when macrophages were stimulated with the Mincle/Mcl ligand TDM, but not when LPS was used (100). The functional consequence of this targeting of Dectin-2 family CLR expression by IL-4/IL-13 has not been addressed to date, but since Mincle/Mcl bind TDM and Dectin-2 has been identified as a PRR for mannose-capped LAM (29), the lack of receptors for recognition of two major mycobacterial PAMPs can be expected to diminish the response of macrophages to encounter with MTB (Figure 2A).

#### Deactivation of Antimicrobial Capacity by IL-10 in Macrophages

IL-10 by several cell types is upregulated during helminth infection (see also above in Section “Direct Regulation of Macrophage Function by Helminths”). Through its powerful macrophage deactivation properties, it inhibits the production of many cytokines important for development of Th1/Th17 responses, such as IL-12, IL-6, IL-1, and IL-23 (101) (Figure 2C). In addition, IL-10 more directly impairs killing of mycobacteria in macrophages by delaying phagosome maturation (102) and inhibiting the expression of IFNγ-induced antimycobacterial effector molecules, such as iNOS and ROS (103). Overexpression of IL-10 in mice makes them more susceptible to mycobacterial infection (104, 105), whereas its deletion or blockade of its receptor lead to enhanced immune control of MTB (106).

## Open Questions Regarding Mechanisms and the Impact for Vaccination Strategies

### Which PRRs Are Regulated by Type 2 Immune Responses in Humans and in Animal Models?

To date, the information about changes in the expression of TLR and CLR during helminth infection is patchy at best, with the observations on TLR2 and TLR9 made in human patients with filarial disease and the effect of IL-4/IL-13 on Dectin-2 family CLR expression in human and mouse macrophages and DC treated *in vitro*. A comprehensive expression analysis of different cell types (monocytes/macrophages, granulocytes, DC) from humans infected with helminths or not, or before and after anthelmintic treatment, should be performed. For comparison, the same type of expression analysis should be done using myeloid cells isolated from mice infected with helminths experimentally. This expression analysis should also be extended to other PRR families, including cytosolic sensors such as NOD proteins, cGAS, and STING.

### Does Impaired Expression of PRR Directly Affect Innate Resistance to MTB Infection?

Reduced expression of receptors for mycobacteria on myeloid cells may impair the antimycobacterial response at several levels. First, cell surface-localized CLR and TLR can be involved in phagocytosis and their absence on the membrane can lead to suboptimal uptake of mycobacteria. While Mincle and Dectin-2 appear to be dispensable for phagocytosis of mycobacteria, Mcl-deficient mice have a delayed clearance of MTB from the airways after experimental infection (37). A lack of TLR-induced signaling during phagocytosis can alter the phagosome maturation process and the capacity for antigen presentation (107, 108), which is also likely in case of CLR deficiency (109, 110). On the other hand, mycobacterial cord factor delays phagosomal acidification in a Mincle-dependent process (111). With regard to adaptive immune responses, the production of Th1- and Th17-inducing cytokines in response to contact with mycobacteria requires TLR-Myd88 and CLR-Syk-Card9 signaling, respectively (23, 24); thus, downregulated expression may alter the quality of the Th cell response toward Th2/Treg and thereby allow mycobacterial growth (Figure 2A). Finally, the capacity of macrophages to kill mycobacteria directly and without further activation by T cells is likely dependent on the strength of activation by the different PRR pathways triggered by MTB. Thus, innate resistance to MTB can be hypothesized to be compromised when the macrophages encountering MTB, such as alveolar macrophages, are exposed to type 2 environment and downregulate receptor expression. On the other hand, to demonstrate that diminished cytokine production and killing of mycobacteria is indeed due to abrogated expression of CLR or TLR, and not to other effects of IL-4-Stat6 signaling, will require to reconstitute expression of these PRR, e.g., by retroviral transduction.

### Does Helminth Infection Hinder Vaccine-Induced Immunity *via* Impaired PRR Expression?

Negative effects of concurrent helminth infection on the immune response to vaccination with BCG have been described repeatedly in mice and humans (65, 66). However, it is unclear by which mechanism helminth-induced type 2 immunity interferes with Th1 response induction by BCG (66). Several studies have demonstrated that helminths can inhibit DC activation and costimulatory molecule expression (112, 113). Helminth infection promotes increased induction of Treg (114, 115) and/or IL-10 production (116), leading to suppression of Th1/Th17 immune responses. Downregulation of TLR and CLR by helminths through a type 2 response may be an alternative mechanism: lack of activating signaling reduces production of cytokines driving Th1/Th17 polarization of immune response. New vaccination strategies using recombinant MTB antigens together with different adjuvants as booster vaccines after BCG priming are currently developed. Among the adjuvants employed in these experimental and preclinical vaccines are the TLR ligands IC31 (TLR9) and the CLR ligands TDB (Mincle/Mcl). Since expression of these receptors is downregulated in patients with helminth coinfection (95, 96) or *in vitro* by type 2 cytokines (100), the adjuvanticity of these vaccines would appear to be particularly vulnerable due to diminished innate activation in helminth-infected patients. To determine whether helminths impair the success of adjuvanted subunit vaccines *via* downregulation of selected PRRs, studies in mouse models should be conducted comparing the efficacy of different adjuvants acting through different innate receptors subject to regulation by helminths or not (e.g., Dectin-1/Curdlan). These studies may also identify optimal adjuvants for use in populations with high rates of helminth infection. Priming of macrophages with the TLR4 ligand LPS can overcome the inhibitory effect of IL-4 on Dectin-2 family CLR expression (100). Thus, the combination of several adjuvants should also be tested because it may circumvent the impaired receptor expression imposed by helminth-induced type 2 responses.

### Can Anthelmintic Treatment Increase Vaccination Efficiency?

The connection between helminth infection and the immune response to tuberculosis and the BCG vaccine has triggered attempts to use anthelmintic treatment (“deworming”) to enhance protective natural or vaccine-induced antimycobacterial immunity. Elias et al. observed that Albendazole increased the Th1 (IFNγ/IL-12) response in adult helminth-infected BCG vaccinees (65, 117). Since most BCG doses are given to neonates, the finding that maternal helminth infection impairs the vaccine-induced IFNγ response after birth was highly relevant (67, 118) and suggested that treatment of mothers before birth should enhance the immune response to BCG in their offspring; however, this was not confirmed in a larger study performed in Uganda (69). Together, the literature is not conclusive yet and larger studies in different settings will be required. Studies into the effect of anthelmintic treatment will also be required for the newly developed live and subunit TB vaccines, depending on how strongly they are affected by coinfection with helminths.

### Can Specific Components of Type 2 Immune Responses Be Targeted to Boost Antimycobacterial Host Responses?

A better understanding of the regulation of protective immunity to TB by helminths at the molecular and cellular level may also contribute to more specific modulation by targeting specific components of the type 2 response. Identification of key factors that suppress Th1/Th17 immunity may allow to specifically antagonize them during vaccination or tuberculostatic treatment without abrogating more beneficial effects of worm-induced immune regulation. It will therefore be important to dissect the contribution of different cell types (e.g., Th2, ILC2, different myeloid cells), cytokines (IL-4/IL-13, IL-10, TGFβ, but also IL-33), and mediators (e.g., Nos2, Arg1), to impaired antimycobacterial resistance and immunity.

## Concluding Remarks

It is now evident that helminth infestations can influence the host response to MTB at multiple levels, from the initial encounter between macrophage and bacilli, to the type of adaptive T cell immunity, and to the development of immunopathology. At the same time, many open questions remain to be answered, both at the clinical–epidemiological level (e.g., regarding the benefit of anthelmintic treatment) and at the fundamental level of immune regulation during coinfection. Clearly, to obtain a mechanistic understanding of observations made in patient cohorts and hypotheses derived from them need to be tested in experimental animal and *in vitro* models. *Vice versa*, it will be exciting to determine whether regulatory effects of type 2 cytokines on macrophages and other cell types identified in laboratory studies hold true also in cohorts of coinfected patients or in vaccination studies in humans.

## Author Contributions

RL and JS conceived and wrote the manuscript and prepared the figures.

## Conflict of Interest Statement

The authors declare that the research was conducted in the absence of any commercial or financial relationships that could be construed as a potential conflict of interest.
